# Supplementary material for: The relationship between family functioning and depression among adolescents in China during the normalization stage of the COVID-19 epidemic: The mediating role of resilience
Source: PLoS One. 2025 May 20;20(5):e0322939. doi: 10.1371/journal.pone.0322939 (PMC12091772; doi:10.1371/journal.pone.0322939)
Supplement: Attachment — Submitted filename: Response to Reviewers .docx [file pone.0322939.s001.docx]

Dear Editors,

We are very grateful that you have taken the time to review our paper. Your feedback is extremely valuable and of great significance for us to improve our work. The following is our detailed response to each of your review comments.

In response to “the individual Jun Zhao named as an investigator on the ethics documents is not an author on the present submission. ” , we have made the following explanations:

Dr. Jun Zhao, the project applicant, has authorized Yang Zhou, the author, to act as the project leader to complete the implementation of this project, and has obtained the consent of the project applicant not to be named as an author.

Thank you again for your professional review, and we look forward to your further feedback.

Sincerely,

Yameng Feng
