# Supplementary material for: The relationship between family functioning and depression among adolescents in China during the normalization stage of the COVID-19 epidemic: The mediating role of resilience
Source: PLoS One. 2025 May 20;20(5):e0322939. doi: 10.1371/journal.pone.0322939 (PMC12091772; doi:10.1371/journal.pone.0322939)
Supplement: Attachment — Submitted filename: Response to Reviewers.docx [file pone.0322939.s002.docx]

**Response to Reviewers**

Dear Editors and Reviewers:

Thank you for your letter and for the reviewers’ comments concerning our manuscript entitled “The relationship between family function and depression among adolescents in China during the normalization stage of the COVID-19 epidemic: the mediating role of resilience” (ID: PONE-D-24-43593). Those comments are all valuable and very helpful for revising and improving our paper, as well as the important guiding significance to our research. We have studied comments carefully and have made corrections which we hope to meet with approval. In the revised manuscript, revised portions are highlighted with red color. The main corrections in the paper and the responds to the reviewer’s comments are as follows.

**Response to Academic Editor's Comments**

**Methods**

1.Selection Bias: The participants are drawn from a single secondary vocational school in Wuhan. This limits the generalizability of the findings. The authors should clarify if these students are representative of adolescents in Wuhan or broader China.

**Response**:

We are extremely grateful for your valuable suggestions. Indeed, the subjects of our study are only from one secondary vocational school in Wuhan. Unlike ordinary high schools, students in secondary vocational schools pay more attention to practical skills rather than theoretical knowledge. This difference in educational models may affect the detection of depression and the magnitude of the indirect effect of psychological resilience between family function and adolescent depression. However, it has little impact on the direction of the relationship between family function and adolescent depression, as well as on whether psychological resilience plays a mediating role.

Based on this, in the "Limitations" section of the revised manuscript, We have provided additional clarification, which is stated as "First, participants were from only one high school, these students cannot fully represent adolescents in Wuhan or broader China, which may limit the generalizability of the findings, larger-scale longitudinal studies from more regions and a wider variety of schools (including vocational high schools and ordinary high schools) should be conducted to further validate our research findings". **(The revised parts are marked in red on lines 320 – 325)**

2. Control of Confounding Variables: While some demographic variables (e.g., sex, smoking, and alcohol use) are controlled, other potentially important factors, such as socioeconomic status and prior mental health conditions, are not addressed.

**Response:** We are extremely grateful for your valuable suggestions, which have great guiding significance for further ensuring the reliability of our research findings. In accordance with your suggestions, we have incorporated the two variables of family economic situation and prior mental health conditions into the analyses. In Table 1, we have added information to show the distribution of PHQ-9 scores and depression detection rate within these two variables. In the structural equation model, we have added control for these two confounding variables, and have also made corresponding corrections to the data in “result” section. **(The revised parts are marked in red in table 1, figure1, table3)**

3. Questionnaire Self-Reporting: Reliance on self-reported data raises concerns about social desirability and recall biases. Discuss it at the end of the manuscript.

**Response:** Thank you for your suggestions. Indeed, in epidemiological surveys, social desirability and recall biases cannot be completely avoided. However, we have taken some necessary measures during the research design and implementation phases to control and mitigate their impacts. Firstly, all the staff involved in this survey are from the Wuhan Mental Health Center and have received unified training. Secondly, during the survey process, all students completed the questionnaires individually to avoid the influence of others, ensuring the authenticity of the data and reducing social desirability. In cases where respondents do not answer seriously or lack the ability to answer, the investigators will take the initiative to communicate directly with them to boost their motivation to answer and the response rate, thereby reducing non-response bias.

We have added a detailed introduction to the control of social desirability and recall biases in the “method” section **(the revised parts are marked in red on lines 127-130)**. Additionally, we'll provide further explanations in the “limitations” section. **(the revised parts are marked in red on lines 325-327)**

**Results**

1.Ambiguity in Statistical Reporting: The presentation of mediation analysis (Table 4, Figure 1) lacks clarity. The text repeatedly emphasizes indirect and direct effects but fails to sufficiently elaborate on their real-world implications.

**Response:** We sincerely appreciate you pointing out the issues regarding Table 4 and Figure 1. Indeed, Table 4, Figure 1 and Table 3 present some overlapping results. After careful consideration by our research team, we've decided to remove Table 3.

In the revised manuscript, figure 1 combined with Table 3 (Table 4 in the original manuscript) could show the main results of our research, figure 1 shows the pathway of key variables (family function, psychological resilience, depression), and it presents the coefficients of the independent variables on each path. Table 3 shows proportion of the direct effects and the indirect effects of family function on depression.

2.Interpretative Overreach: The manuscript concludes that family function has a protective effect against depression via resilience, but the findings are correlational. This should be stated more cautiously. Avoid implying causality in discussions of resilience as a mediator.

**Response:**

We sincerely appreciate your reminder of the language expression issues in our manuscript. Our team has carefully reviewed the original manuscript and, based on your suggestions, modified many parts where the language expressions were not cautious enough. **(the revised parts are marked in red on lines 203-207)**

**Discussion**

1.The discussion assumes that findings are broadly applicable despite the study's limited geographic scope.

**Response:**	We sincerely appreciate your concern regarding the issue of sample representativeness in our research. Indeed, the samples in this study have geographical limitations and may not fully represent high school students in Wuhan, let alone those across the nation. Although the representativeness of the sample may be insufficient, our team believes that it has not fundamentally undermined the core findings and value of this research. That is, family functioning is related to depression, and psychological resilience plays a partial mediating role in this pathway. In the follow-up, we will conduct a study with a larger sample size, involving more schools and covering a broader geographical area, based on the current investigation.

We have provided additional clarification in the “limitations” section. **(the revised parts are marked in red on lines 320-325)**

2.Propose novel, context-specific interventions beyond resilience training: While resilience training is widely recognized, the discussion does not provide innovative strategies or context-specific interventions (e.g., leveraging technology for mental health support).

**Response:** Thank you for your valuable suggestions, which are of great significance for enhancing the value of our research. After carefully reviewing numerous research papers, in the "Mediating role of psychological resilience" part within the "Discussion" section of the revised manuscript, we have listed several innovative strategies and context-specific interventions. **(the revised parts are marked in red on lines 284-299)**

3.Expand on how the findings can inform existing national policies: The paper references national initiatives on adolescent mental health but does not sufficiently engage with how the findings can inform or enhance these programs.

**Response:** Thank you for your valuable suggestions, which are of great significance for enhancing the value of our research. Through thorough contemplation, in the "Mediating role of psychological resilience" part within the "Discussion" section of the revised manuscript, some policies that contribute to enhancing students' psychological resilience have been proposed. **(the revised parts are marked in red on lines 300-306)**

**Response to Reviewer 1's Comments**

**General Comments**

1. Generally congratulation for the hard work on writing this manuscript. However, several issues should be improved: (1) Adhere to JOURNAL guidelines. (2) Work extensively to be clear grammar and typographical errors throughout the document.

**Response:** Thank you for acknowledging our hard work on the manuscript. Our team has conducted a comprehensive review and correction of the original manuscript based on your suggestions and in compliance with journal guidelines. We've ensured that the format of the revised manuscript and the citation of references both meet the journal's specifications. We've carried out strict proofreading to eliminate grammatical and spelling errors. The revised parts have been highlighted in red in the revised manuscript.

**Specific Comments**

**Abstract**

1.On part of method the authors should explain what type of design and approach used also study population.

**Response:** We are extremely grateful to you for pointing out problems of expression in our manuscript. In the abstract part of the revised manuscript, we have improved the description of the research method, which is stated as "A cross-sectional survey was carried out among 2,410 students at a secondary vocational school between September and December 2022 in Wuhan". **(the revised parts are marked in red on lines 26-27)**

2.I noted line no 28 make it clear.

**Response:** We are extremely grateful to you for pointing out problems of expression in our manuscript. In the abstract part of the revised manuscript, we have improved the description of the scales, which is located in line 28. It is now stated as “The Adaptation, Partnership, Growth, Affection, and Resolve Caring Index Questionnaire (APGAR), the 10-Item Connor-Davidson Resilience Scale (CD-RISC-10), and the Patient Health Questionnaire-9 (PHQ-9) were used to evaluate students' family function, psychological resilience and depressive symptoms”. **(the revised parts are marked in red on lines 30-31)**

**Introduction**

1.Introduction well written but I noted to line 107-113 the authors should put it clear in order to remove the confusion to the reader.

**Response:** We are extremely grateful for your valuable suggestions. We have reorganized the statements in this part, and we started a new paragraph to introduce the purpose of this study, stated as “Indeed, several studies have documented that psychological resilience significantly and negatively predicts the onset of depression in adolescents. However, little is known about whether it mediates the relationship between family function and adolescent depression. Therefore, this study aims to explore the external resources (family function) and internal resources (psychological resilience) related to the prevention of depression in Chinese adolescents, and to investigate whether psychological resilience involved in the pathway, so as to provide empirical evidence and a theoretical framework for the development of targeted mental health services for high school students during the post-pandemic period”. **(the revised parts are marked in red on lines 104-112)**

**Method**

1.The authors should give the description of the design also eligibility criteria for participant with sufficient detail to allow replication, including how and when they were actually administered.

**Response:** We are extremely grateful for your valuable suggestions. In accordance with your suggestions, in the revised manuscript, we have added a detailed introduction to the survey methods and the inclusion and exclusion criteria for the participants, which is presented as follows: From January 10th to September 23rd, 2022, a cross-sectional study was performed by Wuhan Mental Health Center and participants were recruited from a secondary vocational school (grades 10–12) in Wuhan by using a cluster sampling method. Inclusion criteria were: (1) 15~18 years old; and (2) could be individually matched with their primary caregivers. Exclusion criteria were: (1) history of neurological, psychiatric, or other serious somatic diseases; (2) unwilling to participate; and (3) nationality was not Chinese. **(****the revised parts are marked in red on lines 115-121)**

2.How sample size was determined? I noted the authors on Sample size calculations are not written the authors should check and make it clear.

**Response:** We sincerely appreciate your concern regarding the sample size. In our study, which employed a cross-sectional design, we determined the sample size using the following formula:

$$n=\frac{z_{a/2}^{2}\times p\times\left( 1-p \right)}{d^{2}}$$

$Z_{\alpha/2}$is the z-score corresponding to the desired confidence level. For a 95% confidence level, $Z_{\alpha/2}\approx1.96$.

Based on previous research, we estimated the prevalence of depression ($p$) to be 0.3.

$d$is the margin of error, which is the maximum amount of error we are willing to tolerate in our estimate of the population mean, we set $d=0.1p$.

Substituting these values into the formula, we calculated the sample size n≈900. Taking non-response and missing data into account, we increased the sample size by 15%. Consequently, the minimum sample size is 1035. Therefore, the sample size of 2500 in our study is sufficient to detect the relationships among family function, psychological resilience, and depression.

3.It’s a protocol this manuscript?

**Response:** This manuscript is not a protocol. It is an original research paper.

**Results**

1.The author should follow the journals guideline on writing the result.

**Response:** Thank you for your suggestions. Our team has conducted a comprehensive review and correction of the original manuscript in compliance with journal guidelines.

**Ethical Consideration**

1.How will you address ethical issue concerning to your study population particularly age 15-18years?

**Response:** For adolescents aged 15-18, we've implemented multiple measures to address ethical concerns. First, we drafted the informed consent form in plain language, fully disclosing the purpose, procedures, and potential benefits and risks of this survey.

Under the principle of voluntary participation, all students must obtain the consent of their legal guardians before they can participate in the survey, and they have the option to withdraw during the survey. The survey was anonymized and the information we obtained would not expose the identities of the participants. **(The revised parts are marked in red on lines 122-132)**

**Limitations**

1.The authors should revise and improve are not clear.

**Response:** Thank you for pointing out the problems in "Limitations" section. After consulting numerous references and careful deliberation, our team has made revisions and supplements to it in accordance with the reviewers' suggestions. **(The revised parts are marked in red on lines 320-327)**

**References**

1.Several references do not fit the requirements of Vancouver style. Revise and improve them.

**Response:** We're extremely grateful to you for pointing out our issues. Our team has reviewed and cross-checked the format of all references to ensure strict compliance with the Vancouver style requirements.

**Response to Reviewer 2's Comments**

**Introduction**

1. Adding reference for line 81, 86, and 100.

**Response:** Thank you for this valuable feedback. In the revised manuscript, we have cited relevant literature at the corresponding positions you mentioned. The reference numbers are 11and 17 respectively, and the citation format meets the Vancouver style requirements. **(The revised parts are marked in red on line 86, 100)**

Reference 11: Manrique-Millones D, Wiium N, Pineda-Marín C, Fernández-Arata M, Alfonso-Murcia D, López-Martínez JL, et al. Association Between Substance Use Behaviors, Developmental Assets and Mental Health: A Glance at Latin American Young College Students. Front Psychol. 2021;12. doi: 10.3389/fpsyg.2021.639578.

Reference 17: Troy AS, Willroth EC, Shallcross AJ, Giuliani NR, Gross JJ, Mauss IB. Psychological Resilience: An Affect-Regulation Framework. Annual Review of Psychology. 2023;74:547-76. doi: 10.1146/annurev-psych-020122-041854.

2. State and describe your problem better.

**Response:** Thank you for pointing out the problems in "Introduction" section. Revisions and supplements have been made in accordance with the reviewers' suggestions. **The revised references are marked in red on lines 104-112.**

**Method**

1. State your study design.

**Response:** We are extremely grateful for your valuable suggestions. In accordance with your suggestions, in the revised manuscript, we have added a detailed introduction to the survey methods and the inclusion and exclusion criteria for the participants, which is presented as follows: From January 10th to September 23rd, 2022, a cross-sectional study was performed by Wuhan Mental Health Center and participants were recruited from a secondary vocational school (grades 10–12) in Wuhan by using a cluster sampling method. Inclusion criteria were: (1) 15~18 years old; and (2) could be individually matched with their primary caregivers. Exclusion criteria were: (1) history of neurological, psychiatric, or other serious somatic diseases; (2) unwilling to participate; and (3) nationality was not Chinese. **(The revised parts are marked in red on lines 115-121)**

2. How did validity for questionnaires done?

**Response:** A series of measures were taken during the design and implementation stages of this survey research to ensure the rigor and accuracy of this study. (1) We consulted with experts in the relevant disciplines before developing and finalizing the questionnaire, and a pilot survey was conducted to further refine the questionnaire. (2) A sample size of over two thousand ensures that the statistical inferences of this study have sufficient power. (3) During the implementation of the survey, all investigators were staff members from Wuhan Mental Health Center and had received unified training. (4) All participants filled out the questionnaires independently, and the investigators respected the wishes of the respondents, avoiding coercion or inducement. (5) In cases where respondents do not answer seriously or lack the ability to answer, the investigators will take the initiative to communicate directly with them to boost their motivation to answer and the response rate. **(The revised parts are marked in red on lines 122-132)**

3. Please write down the code of ethics you received from the institution you mentioned.

**Response:** Thanks for your suggestion. The study protocol was reviewed and approved by the Ethics Committee of Wuhan Mental Health Center, and the code is KY2022.11.01. **(The revised parts are marked in red on line 133)**

4. Dedicate a section in methodology for ethical approval and inform consent.

**Response: Thanks for your suggestion**. In the revised manuscript, we have supplemented the introduction of ethical approval and informed consent, expressed as “Before the formal survey, informed consent forms were distributed to students and their legal guardians, all students must obtain the consent of their legal guardians before they can participate in the survey”. **(The revised parts are marked in red on line 123-126, 132-133)**

5. Explain the chi square test.

**Response:** Chi-square test is used to test whether there are statistically significant differences in depression detection rate among different demographic characteristics. **(The revised parts are marked in red on line 164-166)**

6. How did you determine the level of depression symptom?

**Response:** We determined the level of depression symptoms based on Patient Health Questionnaire-9 (PHQ-9). PHQ-9 is a commonly used tool for assessing the severity of depressive symptoms and has been widely applied and recognized worldwide. Its scoring criterion is to add up the scores of the 9 questions to obtain the total score, which ranges from 0 to 27 points. Higher total scores indicate more severe depression. Those with a total score greater than 4 points were determined to have depressive symptoms.

7. Explain the structural equation modeling technique more clearly.

**Response:** Thank you for your suggestions. In the revised manuscript, we have supplemented the introduction of structural equation model, expressed as “A structural equation model (SEM) was constructed using the PROCESS macro program in SPSS 22 to examine the mediating effect of psychological resilience on the relationship between family functioning and depression. In SEM, the root mean square error of approximation (RMSEA) value is <0.08, and comparative ﬁt index (CFI) and Tucker Lewis index (TLI) values are >0.90, indicating acceptable models. All statistical tests were two-sided and P<0.05 was considered statistically signiﬁcant”. **(The revised parts are marked in red on line 167-172)**

8. Provide a normality test and explain whether your data is normal or not.

**Response:** Thank you for your suggestion. A normality test was conducted, depression score evaluated by PHQ-9 did not conform to a normal distribution are expressed as the median and interquartile range (M (Q25, Q75)), **the revised parts are marked in red in table1.** The scores for psychological resilience, family function did not conform to a normal distribution, Spearman correlation analysis was performed for psychological resilience, family function and depression, **the revised parts are marked in red in table2.**

**Results**

1. I suggest using regression tests for predicting factors.

**Response:** Thank you for suggesting. The aims of our study were to explore the external resources (family function) and internal resources (psychological resilience) related to the prevention of depression in Chinese adolescents, and to investigate whether psychological resilience involved in the pathway. Correlation analysis and structural equation modeling are the main statistical analysis methods for achieving the research objectives. Since the aim of our study is not to explore whether family function and psychological resilience can predict the occurrence of depression, we did not use the regression model for prediction in this research.

Our research team would like to express our sincere apologies for the inaccurate expressions in the “results” section, which led you to misinterpret that we aimed to use correlation analysis to predict the occurrence of depression. We have already revised these imprecise statements. **(The revised parts are marked in red in lines 203-207)**

**Discussion**

1. There's no need to divide the discussion into sub-sections.

**Response:** Thank you for your suggestions. We divided the discussion into sub-sections for several important reasons. Firstly, each sub-section is dedicated to a specific theme, we can systematically address different aspects of our research findings. Secondly, separating the discussion into sub-sections helps in maintaining a clear and logical flow. It prevents the discussion from becoming a jumbled mess of ideas, ensuring that each point is developed thoroughly and coherently. Finally, this approach improves the readability of the manuscript. For readers interested in a particular aspect of our research, they can quickly jump to the relevant sub-section.

We believe that the sub-sections in the discussion section are crucial for a comprehensive and accessible presentation of our research.

**Figures and tables**

1. Provide a table for descriptive data and status of depression symptoms.

**Response:** Thanks for your valuable suggestions. We have added new content to Table 1 of the revised manuscript to describe the population distribution of depression score which was evaluated by PHQ-9. Ultimately, Table 1 can present the distribution of depression score and the detection rate of depression. **(The revised parts are marked in red in table1)**

**Some more issues should be considered necessary for publication**

1. Suggestions for future studies also be mentioned.”

**Response:** Thanks for your valuable suggestions. In the revised version, we've added a suggestions for future studies in “limitations” section. We suggest that some larger-scale longitudinal studies from more regions and a wider variety of schools (including vocational high schools and ordinary high schools) should be conducted **(The revised parts are marked in red in lines 320-325)**
